# Viral Infection in Esophageal, Gastric, and Colorectal Cancer

**DOI:** 10.3390/healthcare10091626

**Published:** 2022-08-26

**Authors:** Takeshi Yamashina, Masaaki Shimatani, Masahiro Takeo, Kotaro Sasaki, Masahiro Orino, Natsuko Saito, Hironao Matsumoto, Takeshi Kasai, Masataka Kano, Shunsuke Horitani, Kimi Sumimoto, Toshiyuki Mitsuyama, Takafumi Yuba, Toshihito Seki, Makoto Naganuma

**Affiliations:** 1 Department of Gastroenterology and Hepatology, Kansai Medical University Medical Center, Moriguchi 570-8507, Osaka, Japan; 2 Division of Liver Disease Center, Kansai Medical University Medical Center, Moriguchi 570-8507, Osaka, Japan; 3 The Third Department of Internal Medicine, Kansai Medical University, Hirakata 573-1010, Osaka, Japan

**Keywords:** viral infection, gastrointestinal tract cancer, virome, bacteriophages, esophageal cancer, gastric cancer, colorectal cancer

## Abstract

The human gastrointestinal tract, which constitutes the digestive system, contains a large number of virus particles that maintain organizational homeostasis and health. Conversely, viral pathogens have also attracted attention for their involvement in the pathogenesis of certain cancers, including gastrointestinal cancers. To aid prevention and treatment of these cancers, the relevance of gastrointestinal viral factors as potential risk factors needs to be carefully investigated. This review summarizes and discusses the available literature on the relationship between the development of esophageal, gastric, and colorectal cancers and their corresponding viruses. This review reveals that research on the association between colorectal cancer and viruses, in particular, is still in its infancy compared to the association between HPV and esophageal cancer and between EBV and gastric cancer.

## 1. Introduction

Gastrointestinal (GI) tract cancers are one of the most common groups of cancers in terms of global incidence (colorectal cancer is the third most commonly occurring cancer, while stomach cancer is fifth and esophageal cancer is seventh). They also have high mortality rates, with colorectal cancer being the second most common in terms of mortality, followed by stomach cancer and cancer of the esophagus in fourth and sixth place, respectively. More than 4.2 million new GI tract cancer cases and more than 2.2 million deaths were estimated to occur in 2020 [1].

The mechanism of carcinogenesis of GI tract cancers is considered to be varied and dependent on a number of factors. Infectious diseases are reportedly responsible for up to 50% of carcinogenesis in humans, with viruses in particular accounting for 10% to 15% of cases [2]. In recent years, the microbiome, mainly comprising the bacteriome and virome, has been highlighted as having an impact on carcinogenesis. The terms bacteriome and virome are mostly used to describe the assembly of bacteria and viruses present within the healthy human GI tract, and the genetic information of such organisms. Importantly, the microbiome also has a major influence on the immune system [3,4]. Toxic changes in the microbiome can induce carcinogenesis through the growth of carcinogenic bacteria and the effects of bacterial metabolites on the host [5,6]. Even though the bacteriome and the virome interact, most research so far has focused on the bacteriome with less being conducted on the virome. This review summarizes and discusses the current knowledge regarding the relevance of viruses in the development of cancers of the digestive system.

## 2. The Virome

Many viruses are present in healthy humans, being found mainly in the GI tract of the human body, and are collectively referred to as the human virome. The human virome comprises viruses that can infect human cells, and bacteriophages, which infect live bacteria and are the most common group of environmental viruses.

A large number of viral particles are present in the human GI tract and related organs that make up the digestive system [7]. The virome contributes to maintenance of intestinal homeostasis; for example, Kernbauer et al. demonstrated that infection with the murine norovirus (MNV) restored intestinal morphology and lymphocyte function in germ-free or antibiotic-treated mice without causing overt inflammation or illness [8] and Thépaut et al. reported infection with MNV improves survival in mice with *Pseudomonas aeruginosa* acute lung damage and reduces pro-inflammatory cytokine production in vivo [9]. In contrast, changes in the virome have already been shown to be involved in a variety of diseases, such as inflammatory bowel disease and colorectal cancer [10,11]. Disturbances in the steady state of the human virome can lead to intestinal inflammation and have also been reported to be associated with inflammatory bowel diseases, such as Crohn’s disease and ulcerative colitis [12,13,14]. It has also been suggested that the virome may become altered in a way that promotes carcinogenesis [11,15].

### Bacteriophages

**Figure 1:**
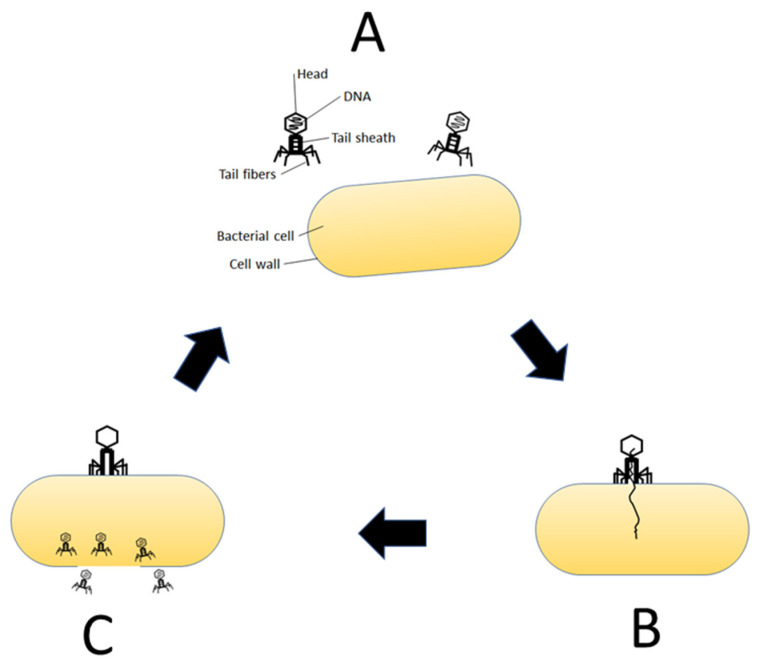
(**A**) Bacteriophage replication starts with host recognition and absorption. (**B**) Attachment of phage to the bacterial cell and injection of the phage DNA. (**C**) Synthesis of phage DNA using bacterial materials and release of progeny phage.

Bacteriophages were first discovered in 1915 and were then identified as a factor in the bacteriolysis of *Staphylococci* and *Shigella* in 1917. Bacteriophage is the general term for viruses that can infect bacterial cells and are widespread in the GI tract. The majority of viruses in the GI tract microbiome are regarded as bacteriophages [16]. Bacteriophage replication requires a live bacterial host, resulting in lysis of the host cell and release of progeny phage (Figure 1). This is primarily known as the lytic cycle or lysogenic cycle. In recent years, much attention has been focused on bacteriophage therapy [17] and food contamination by bacteriophages [18], because they are a major microbiological threat to fermented food manufacturing, but have also been recognized as promising antimicrobial agents for controlling certain bacterial pathogens in food production. Bacteriophage therapy is a method of treating bacterial infections by causing viruses to multiply inside the bacterial cells, followed by lysis of the cells and elimination of the bacteria. Thus, bacteriophages can affect the composition and dynamics of the human virome.

## 3. Cancers of the GI Tract

### 3.1. Esophageal Cancer

The main histological types of esophageal cancer are esophageal squamous cell carcinoma (ESCC) and esophageal adenocarcinoma (EAC). The major risk factor for ESCC is acetaldehyde from alcohol consumption, but smoking is also important [19]. Another possible factor is poor nutritional status and vitamin deficiencies [20]. The main risk factors for EAC are Barrett’s esophagus and obesity [21]. However, in recent years, an association between viral infections and esophageal cancer, mainly ESCC, has also been identified.

Human papillomavirus (HPV) infection is considered to be the cause of most cases of cervical cancer [22], and has also been associated with cancers of the cervix, penis, vulva, vagina, anus, and oropharynx [23]. Similar to these squamous cell carcinomas, HPV is also suspected to be associated with ESCC carcinogenesis. Petrelli et al. reported that HPV is detectable in approximately 1 in 5 cases of ESCC with different geographical prevalence [24]. Regarding these regional differences, the prevalence of HPV in esophageal cancer is particularly high in Asia [24,25]. The pathogenesis of HPV-induced transformation of normal squamous cells into cancer cells is not fully known, although oncogenic HPV E6 and E7 genes promote ESCC pathogenesis by upregulating susceptible human leukocyte antigen-DQB1 via DNA demethylation [26]. EAC and Barrett’s esophagus have also been linked to HPV infection, but only in a small number of reports and some cases are HPV-negative [27]. According to Rajendra et al., the active involvement of HPV in Barrett’s dysplasia and EAC is characterized by abnormalities in the wild-type p53 and retinoblastoma protein pathways using fresh frozen tissue [28]. Further studies comprising a large number of cases and taking into account regional characteristics of both EAC and ESCC are needed.

Hepatitis C virus (HCV) is a human pathogen that causes liver diseases and extrahepatic complications, such as cardiovascular events and neurological manifestations. In addition, HCV has recently been reported to be associated with many extrahepatic malignancies, including esophageal cancer [29]. Furthermore, a recent meta-analysis showed a modest positive association between esophageal cancer and chronic HCV infection with a pooled relative risk of 1.61 (95% confidence interval [CI], 1.19–2.17) [30]. However, this meta-analysis failed to distinguish between EAC and ESCC, and therefore further study is needed. In general, most reports of ESCC are from Asia, especially Japan, while most reports of EAC are from Europe; treatment for ESCC and EAC could be different. There is a need for additional research involving numerous cases and accounting for regional EAC and ESCC features.

### 3.2. Gastric Cancer

The most common histological type of gastric cancer is adenocarcinoma, which accounts for approximately 95% of all malignant tumors of the stomach [31]. Risk factors for gastric cancer are known to be *Helicobacter pylori* infection, age, male sex, smoking, race, pharmacological treatment, radiation, low levels of physical activity, high intake of salty foods, low intake of fiber, and genetic background [32]. Although various factors, including bacterial, host, and environmental factors, are thought to interact in the carcinogenesis of gastric cancer, chronic inflammation caused by *H. pylori* infection is considered to be the most important [33]. However, because only 3% of *H. pylori*-infected patients develop gastric cancer [33], it is possible that other factors may be also involved in the development of gastric tumors.

Epstein–Barr virus (EBV) is the most well-known virus involved in gastric adenocarcinoma and approximately 10% of cases are estimated to be EBV-associated [34]. EBV is known to be associated with various tumors and immune diseases. For example, EBV DNA was detected in 60% of synovial fluids in rheumatoid arthritis patients, suggesting a possible role for EBV in the pathophysiology of rheumatoid arthritis as an autoimmune disease [35]. EBV-associated gastric cancer is characterized as more common in male patients, with high prevalence in the upper part of the stomach and multiplicity. Several factors have been implicated in EBV-induced gastric carcinogenesis, including hypermethylation of tumor suppressor genes, inflammatory changes in the gastric mucosa, host immune evasion by EBV, and alterations in cell cycle pathways [34]. However, Aversa et al. reported low EBV prevalence in gastric adenocarcinoma among a high-incidence population in China [36]. Previous reports have shown a particularly high positivity rate in gastric cardia localization [37], but Aversa et al. reported a rate of only 0.9% in cardia localization. Several studies have shown that patients with EBV-associated gastric cancer may have a good prognosis [37,38], as well as their response to immunotherapy [39,40]. The reasons for this are not yet clear, but it is thought that tumor-infiltrating lymphocytes may enhance cell-mediated cytotoxicity and may be more sensitive to chemotherapeutic agents [41]. There are still many unknowns regarding the carcinogenesis of gastric cancer caused by EBV, with further research into the carcinogenic mechanism required.

It is well known that hepatitis B virus (HBV) infection causes hepatocellular carcinoma, and there have also been several reports of its involvement in gastric cancer [42,43,44]. Wei et al. demonstrated that HBV DNA was detected in 12.4% of gastric cancer tissues and identified HBV core antigen commonly in lymphocytes in gastric cancer tissues by immunohistochemical staining [42]. A meta-analysis by Chui et al. also found that HBV infection increased the risk of gastric cancer (odds ratio (OR) = 1.23; 95% CI, 1.10–1.37) [45]. They also demonstrated that gastric cancer patients who are HBV carriers have lower expression of PD-L1 and may not respond to immune checkpoint blockade therapy [45].

In recent years, chronic HCV infection, as well as HBV, has been associated with gastric malignancies. Huang et al. demonstrated in their large cohort study that patients without sustained virological response (SVR) in HCV infection had a significantly higher risk of gastric cancer, and achieving SVR could also reduce the risk of gastric cancer in patients with chronic HCV infection [46]. However, the potential link between HCV and gastric cancer has not been well documented and a clear pathophysiological mechanism requires further investigation.

Although human cytomegalovirus (HCMV) is well known for its tendency to cause disease in immunocompromised patients, its role in the pathogenesis of cancer has also recently been studied. Wang et al. conducted a meta-analysis using 5 studies and demonstrated that the pooled HCMV prevalence in GC was 43.1% and the pooled ORs showed that HCMV infection was highly associated with the risk of gastric cancer [47]. Regarding the role of HCMV in oncogenesis, a concept called oncomodulation, which promotes tumor progression and spread, has been proposed [48,49,50]. HCMV produces over 250 different proteins in infected cells. These proteins may be involved in the development of carcinogenesis, tumor progression and metastasis [48,49,50]. While the theory of oncomodulation can be applied to some of the associations between HCMV infection and tumors, this concept cannot explain all of the biological observations in HCMV-positive tumors [47].

In addition to EBV, HBV, HCV, and HCMV, there may be other viruses that play a potential role in the development of gastric carcinomas. Wang et al. demonstrated in their meta-analysis that HPV and John Cunningham virus (JCV) are both associated with a statistically significantly increased risk of gastric cancer [47], suggesting that infection with these viruses may be associated with an increased risk of gastric cancer. The biological mechanisms of such virus association with gastric cancer remains subject to further investigation. However, the gastric mucosa, contrary to the esophagus and large intestine, sometimes has a background of inflammation, mainly due to *H. pylori* infection and acid secretion, which may complicate matters.

### 3.3. Colorectal Cancer

Colorectal cancer (CRC) is the third most common cancer worldwide in terms of incidence. In particular, it is the third most common cancer in men and the second most common in women, with the second highest overall rate of mortality. It is estimated that more than 1.9 million new cases of colorectal cancer and 935,000 deaths occurred in 2020 [1]. Multiple factors contribute to the development of colorectal cancer, but early detection and treatment can lead to a good prognosis. Many epidemiological studies have shown that the lifestyle and environmental factors that cause CRC include heredity, high red meat intake, and cigarette smoking [51,52,53]; however, these factors are insufficient to explain the high rate of incidence.

The human colorectum contains a vast number of viral particles that maintain intestinal homeostasis. Alterations in the virome have been implicated in a variety of diseases, and have also been reportedly associated with inflammatory bowel diseases [12,13,14]. Such virome disruptions may be risk factors for developing CRC, especially in terms of JCV, HPV, and HBV.s

JCV was recently classified as a probable class 2B, biological carcinogen in humans by the International Agency for Research on Cancer [54]. Mou et al. reported that JCV DNA positivity was 40.9% (56/137) in colorectal tumor tissues of CRC patients, while Enam et al. reported an incidence of 81.4% (22/27) [55,56]. JCV has been reported to have the potential to promote colon carcinogenesis in a variety of ways. The genome of JCV encodes a transforming protein, T-antigen, which is thought to be involved in the oncogenic properties of the virus and can interact with p53 and pRB tumor suppressor proteins as well as other major signaling pathways [57].

Damin et al. demonstrated an increase in CRC risk with HPV positivity (OR = 10.04; 95% CI, 3.7–27.5) [58] and Baandrup et al. also demonstrated in their meta-analysis that the prevalence of HPV was 36.8% (95% CI, 21.3–53.8%) in adenocarcinomas and 1.6% (95% CI, 0.0–9.6%) in control patients, while the OR for the association between HPV and CRC was 6.0 (95% CI, 2.0–17.9%) [59]. These results indicate an association between HPV infection and CRC risk. However, Chen et al. questioned these results and determined that a meta-analysis could not be done because of the large heterogeneity between studies [60]. Published evidence on the etiology of HPV and CRC is limited and there remains a strong need for large, rigorous epidemiological studies.

Studies of the association between EBV and CRC are also limited, and the results are controversial. Song et al. reported that of 90 CRC specimens, EBV exons and fragments were detected in 27.7% and 32.2%, respectively, which was significantly higher than the 4.0% EBV gene positivity rate in 25 adjacent non-cancerous specimens (*p* < 0.001) [61]. Fiorina et al. also reported that EBV genomic DNA fragments were found in a significant portion of tumors (23/44, 52%) [62]. In contrast, EBV DNA was detected in only 1 of 70 colorectal adenocarcinoma tissues (1.42%) in a study by Sarvari et al. [63]. This discrepancy may be due to differences in the methods used to isolate the genetic material or regional differences in EBV infection rates, demonstrating that while some studies may indicate an indirect role for EBVs in CRC, conclusive evidence has yet to be presented. In colorectal cancer, the research is still less advanced than the association of EBV in gastric cancer or HPV in esophageal cancer.

## 4. Conclusions

In this article, we have described the relationship between particular viruses and the development of gastrointestinal cancers, including esophageal, gastric, and colon cancers (Table 1). Although these relationships have received much attention in recent years, the components of the virome are still largely unknown, and we do not yet have a good understanding of how they affect the risk of developing cancer of the digestive tract. Therefore, studies on carcinogenic mechanisms with standardized sampling and analysis methods and rigorous multicenter observational cohort studies are expected to be helpful in establishing prevention, diagnosis, and treatment methods for these gastrointestinal cancers.

**Table 1:** Overview of the relevant studies for this review investigating potential associations between viruses and esophageal, gastric, and colorectal cancers.

Author	Year	Cancer Type	Virus	Reference Number
Petrelli F	2021	ESCC	HPV	[24]
Petrick JL	2014	ESCC	HPV	[25]
Feng B	2014	ESCC	HPV	[26]
Rajendra S	2020	EAC	HPV	[27]
Rajendra S	2017	EAC	HPV	[28]
Chu Y-Y	2021	EC	HCV	[29]
Ponvilawan B	2021	EC	HCV	[30]
Naseem M	2017	GC	EBV	[34]
Mahabadi M	2016	GC	EBV	[35]
Aversa JG	2021	GC	EBV	[36]
Constanza CM	2014	GC	EBV	[37]
van Beek J	2004	GC	EBV	[38]
Kim ST	2018	GC	EBV	[39]
Xie T	2020	GC	EBV	[40]
Derks S	2016	GC	EBV	[41]
Wei XL	2017	GC	HBV	[42]
An J	2018	GC	HBV	[43]
Mahale P	2019	GC	HBV	[44]
Cui H	2020	GC	HBV	[45]
Huang CF	2020	GC	HCV	[46]
Wang H	2020	GC	HBV, HCMV, HPV, JCV	[47]
Mou X	2012	CRC	JCV	[55]
Enam S	2002	CRC	JCV	[56]
Hampras SS	2014	CRC	JCV	[57]
Damin DC	2013	CRC	HPV	[58]
Baandrup L	2014	CRC	HPV	[59]
Chen H	2015	CRC	HPV, human polyomaviruses, human herpesviruses, human bocavirus, Inoue–Melnick virus	[60]
Li BS	2006	CRC	EBV	[61]
Fiorina L	2014	CRC	EBV	[62]
Sarvari J	2018	CRC	EBV, HPV, HBV, Human polyomaviruses	[63]

ESCC, esophageal squamous cell carcinoma; EAC, esophageal adenocarcinoma; EC, esophageal cancer; GC, gastric cancer; CRC, colorectal cancer; HPV, human papillomavirus; HCV, hepatitis C virus; EBV, Epstein–Barr virus; HBV, hepatitis B virus; HCMV, human cytomegalovirus; JCV, John Cunningham virus.
